# Hypoglycemic Effect of Macrocyclic Binuclear Oxovanadium (IV) Complex on Streptozotocin-Induced Diabetic Rats

**DOI:** 10.1080/15438600490277842

**Published:** 2004

**Authors:** B. Ramachandran, D. Sathish Sekar, M. Kandaswamy, V. Narayanan, S. Subramanian

**Affiliations:** 1 Department of Biochemistry and Molecular Biology University of Madras Tamil Nadu Guindy Campus Chennai 600 025 India; 2 Department of Inorganic Chemistry University of Madras Guindy Campus Chennai India

## Abstract

Though vanadium complexes mimic the action of insulin, owing to their toxicity, research is still in progress for a new vanadium complex with maximum efficacy at low concentration and without any side effects. A novel macrocyclic binuclear oxovanadium complex was synthesized, its composition and structure were confirmed by spectral studies and its efficacy was studied in streptozotocin-induced diabetic rats over a period of 30 days. The oral administration of the complex normalizes the blood glucose level in the diabetic rats and also maintains normoglycemia after a glucose load. The biochemical studies revealed that the complex is not toxic to the system. The nontoxic nature of this complex may be due to the presence of the vanadyl ions in an intact form. The study highlights the nontoxic and hypoglycemic effects of the new macrocyclic binuclear oxovanadium complex.


*Experimental Diab. Res.*, 5:137–142, 2004
Copyright © Taylor and Francis Inc.
ISSN: 1543-8600 print / 1543-8619 online
DOI: 10.1080/15438600490277842

Taylor & Francis
Taylor & Francis Group

### Hypoglycemic Effect of Macrocyclic Binuclear Oxovanadium (IV) Complex on Streptozotocin-Induced Diabetic Rats

**B. Ramachandran,[1] D. Sathish Sekar,[1] M. Kandaswamy,[2] V. Narayanan,[2] and S. Subramanian[1]**

[1]*Department of Biochemistry and Molecular Biology, and* [2]*Department of Inorganic Chemistry, University of Madras, Guindy Campus, Chennai, India*

**Though vanadium complexes mimic the action of insulin, owing to their toxicity, research is still in progress for a new vanadium complex with maximum efficacy at low concentration and without any side effects. A novel macrocyclic binuclear oxovanadium complex was synthesized, its composition and structure were confirmed by spectral studies and its efficacy was studied in streptozotocin-induced diabetic rats over a period of 30 days. The oral administration of the complex normalizes the blood glucose level in the diabetic rats and also maintains normoglycemia after a glucose load. The biochemical studies revealed that the complex is not toxic to the system. The nontoxic nature of this complex may be due to the presence of the vanadyl ions in an intact form. The study highlights the nontoxic and hypoglycemic effects of the new macrocyclic binuclear oxovanadium complex.**

**Keywords** Macrocyclic Complex; Streptozotocin Diabetes; Vanadium

Vanadium has been recognized as an essential nutritional requirement in higher animals. It seems to be a trace element required for normal growth and development and also necessary for the growth and survival of mammalian cell culture [1]. Srivastava [2], Brichard and Henquin [3], Poucheret and colleagues [4], Schechter and Karlion [5], and Shafrir and colleagues [6] have extensively reviewed the antidiabetic effects of vanadium salts, showing that administration of vanadium salts improves glucose tolerance, lowers blood glucose levels, and corrects the metabolic deficiencies in several species of diabetic animals and human patients. Thus, vanadate ions were shown to mimic all or most of the actions of insulin in intact cell systems, via a postreceptor mechanism [7–9].

Heyliger and colleagues [10] observed that vanadate normalized the elevated blood glucose and depressed cardiac performance of diabetic rats. Administration of vanadyl sulphate to streptozotocin-diabetic rats resulted in a persistent normoglycemia [11]. Vanadyl (IV) complexes, such as vanadyl cysteine methyl ester, vanadyl maloante, vanadyl tartarate, and vanadyl salicylaldehyde, normalized diabetic state in alloxan-diabetic rats similarly to vanadate [12]. McNeill and colleagues [13] reported that administration of bis-maltolato oxovanadium (IV) complex (5.8 mM) to streptozotocin (STZ)-diabetic rats normalized blood glucose, lipid, and food and fluid intake without increasing the insulin level. Further, other complexes such as bis-glycinato oxovanadium (IV) complex [14] and bis-(pyrrolidin-*N*-carbodithiato) vanadium (IV) complex [15] were also found to be effective in STZ-diabetic rats.

Though vanadium possesses a range of diabetes-corrective actions, its toxicity is of more concern in rodents and humans [16]. In most studies of the insulin-like effects of vanadium in diabetic rats and mice, oral administration of vanadium was considered to be toxic and the toxicity increased with increasing concentration of vanadium [17, 18]. The use of various

Received 30 May 2003; accepted 3 October 2003.
The authors thank University Grants Commission, New Delhi, India, for their financial assistance to this project.
Address correspondence to Dr. S. Subramanian, Department of Biochemistry and Molecular Biology, University of Madras, Guindy Campus, Chennai 600 025, Tamil Nadu, India. E-mail: subbus2020@yahoo.co.in chelating agents and ligands to reduce vanadium toxicity and improve insulin potency are the major recent goals of vanadium research. In this aspect, vanadium complex with macrocyclic ligand to reduce its toxicity was formulated and its hypoglycemic efficacy was determined in the present study.

### MATERIALS AND METHODS

### Synthesis of Macrocyclic Binuclear Oxovanadium (IV) Complex [19]

*Synthesis of Precursor Compound: 6,6′-piperazine-1,4 diylmethylene-bis (4-methyl-2-formyl phenol)*

A mixture of piperazine, paraformaldehyde and 4-methyl-2-formyl phenol (1:2:2) in ethanol acetic acid medium was stirred for 6 hours at 60°C. The reaction mixture was cooled to room temperature and neutralized with solid sodium carbonate. Ethanol was removed by distillation under reduced pressure and the residue was extracted with chloroform. Removal of chloroform by distillation yielded a pale yellow crystalline solid, which was further purified by silica gel column chromatography.

*Synthesis of Macrocyclic Binuclear Oxovanadium (IV) Complex*

The precursor compound was dissolved in 20 mL of chloroform. To this solution, one equivalent of oxovanadium (IV) sulphate, dissolved in 50 mL methanol, was added. The resulting yellow reaction mixture was refluxed for about 30 minutes. To the above resulting yellow-colored solution, 1 equivalent of oxovanadium (IV) acetylacetonate and 1 equivalent of ethylenediamine were added and refluxed for 1 hour. On slow evaporation of the solvent at room temperature, a dark brown–colored compound was obtained, which was washed with ether and dried in a vacuum dessicator. The complex was recrystallized from methanol. The complex was characterized by elemental analysis, and ultraviolet (UV)-visible, infrared (IR), and electron spin resonance (ESR) spectroscopies (Figure 1). The complex was administered to rats as an aqueous suspension.

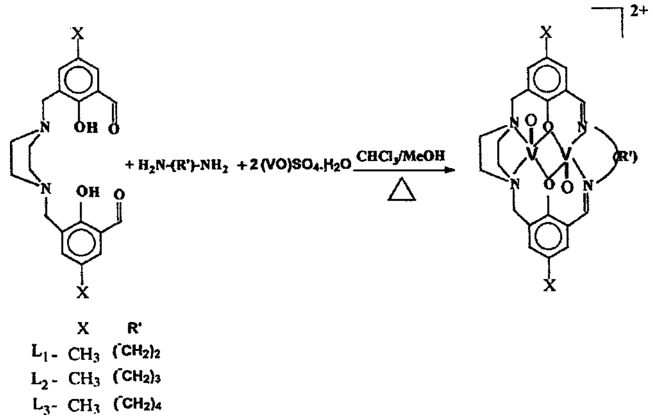

**FIGURE 1**
Synthesis of macrocyclic binuclear oxovanadium complex.

### Animals

Male albino rats of the Wistar strain, weighing around 160 to 180 g, were purchased from Tamil Nadu Veterinary and Animal Sciences University, Chennai, for the present study. They were acclimatized to animal house conditions, fed with commercial pelleted rat chow (Hindustan Lever Ltd., Bangalore), and had free access to water. The experiments were designed and conducted in accordance with the ethical norms approved by Ministry of Social Justices and Empowerment, Government of India, and Institutional Animal Ethics Committee guidelines.

### Experimental Protocol

Animals were fasted for 24 hours before inducing diabetes with STZ. Animals were anesthetized with ether and were intraperitoneally administered with freshly prepared solution of streptozotocin (55 mg/kg body weight) in 0.1 M citrate buffer (pH 4.5) [20]. After 48 hours, rats with blood glucose levels above 250 mg/dL were considered as diabetic rats and were used for the present study. Control animals received citrate buffer alone. The normal and overt diabetic rats were grouped as follows:

Group I: Control rats administered with saline
Group II: Vanadium complex administered control rats (Aqueous suspension of 5 mg/kg body weight/mL/day orally for 30 days)
Group III: STZ-diabetic rats
Group IV: STZ-diabetic rats treated with vanadium complex (Aqueous suspension of 5 mg/kg body weight/mL/day orally for 30 days)

After 30 days of treatment, the rats were euthanized and blood was collected using EDTA as anticoagulant. The whole blood was used for the estimation of glucose [21], urea [22], hemoglobin [23], and glycated hemoglobin [24]. The plasma was used for the assay of cholesterol [25], creatinine [26], proteins [27], glutamate oxaloacetate transaminase (GOT), and glutamate pyruvate transaminase (GPT) [28].

### Glucose Tolerance Test

After 4 weeks of treatment (on the 29th day), fasting blood sample was taken from all the groups of rats. Four more blood samples were collected at 30-, 60-, 90-, and 120-minute intervals [29] after administration of glucose at a concentration of 2 g/kg body weight [30]. All the blood samples were collected

**TABLE 1**
Effect of vanadium complex on blood sugar level in glucose-loaded normal and experimental groups of rats

| Group | Blood sugar level (mg/dL) Fasting | 30 min | 60 min | 90 min | 120 min |
|---|---|---|---|---|---|
| Normal control | 81 ± 5.7 | 147 ± 8.3 | 169 ± 9.2 | 121 ± 8.9 | 85 ± 6.1 |
| Vanadium control | 79 ± 5.3 | 149 ± 8.5 | 165 ± 9.1 | 116 ± 7.8 | 81 ± 5.8 |
| Diabetic control | 254 ± 11.9* | 319 ± 18.9* | 391 ± 19.5* | 350 ± 17* | 315 ± 18* |
| Diabetic, vanadium-treated | 93 ± 6.8* | 152 ± 9.1* | 177 ± 10.1* | 125 ± 9.8* | 98 ± 8.9* |

*Note.* Values are given as mean ± SD for groups of 6 animals in each group.
Values are statistically significant at $^{*}P < .05$. Vanadium-treated control rats were compared with control rats. Diabetic control rats were compared with control rats. Vanadium-treated diabetic rats were compared with diabetic control.

with potassium oxalate and sodium fluoride solution for the estimation of glucose.

### Statistical Analysis

All the grouped data were statistically evaluated with SPSS/7.5 software. Hypothesis testing methods included 1-way analysis of variance (ANOVA) followed by least significant difference (LSD) test. *P* values of less than .05 were considered to indicate statistical significance. All the results were expressed as mean ± SD for 6 animals in each group.

### RESULTS

Changes in body weight gain at regular intervals during the experimental period are shown in Figure 2. The body weight was found to be increased in both normal controls (group I) and

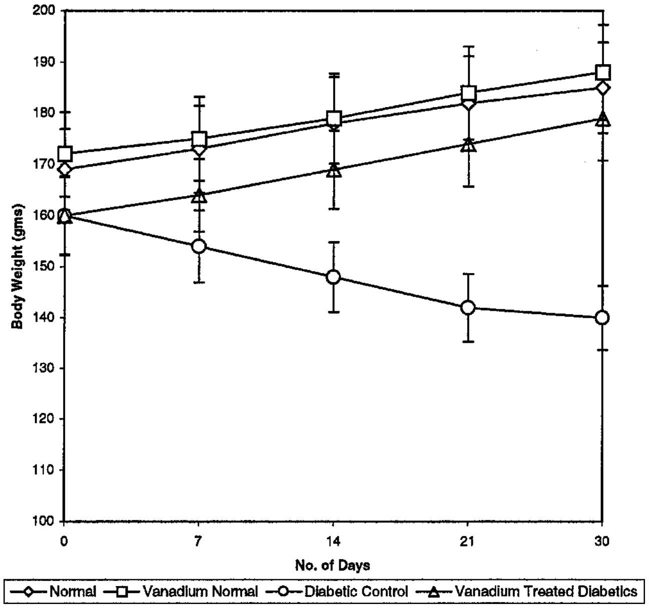

**FIGURE 2**
Change in body weight gain of normal and experimental groups of rats.

vanadium-treated control group of rats (group II). The reduction in body weight in STZ-diabetic rats (group III) were restored to near normal in diabetic rats treated with vanadium complex (group IV).

Table 1 shows the blood glucose levels of normal and experimental groups of rats after oral administration of glucose. In vanadium complex–treated control rats, the change in blood glucose levels was in par with that of the normal rats. In diabetic control rats, the peak increase in blood glucose concentration was observed after 1 hour and it remained high over the next hour. Vanadium complex–treated diabetic rats showed significant decrease in blood glucose concentration at 1 hour and 2 hours when compared with diabetic control rats. The glucose tolerance effect was more pronounced after a 2-hour interval.

Table 2 shows the levels of blood glucose, plasma cholesterol, total proteins, and urine sugar in all the groups of rats. The increase in the levels of glucose and cholesterol and a concomitant decrease in the levels of plasma proteins in diabetic rats were restored to near normal levels in vanadium complex–treated diabetic rats. Urine glucose present in diabetic rats was absent in vanadium complex–treated diabetic rats. No significant alterations were observed in vanadium complex–treated control rats.

Figure 3 shows the levels of hemoglobin and glycated hemoglobin in normal and experimental groups of rats. The increase in the levels of glycated hemoglobin and a corresponding decrease in the levels of hemoglobin found in diabetic rats were restored to near normal in vanadium complex–treated diabetic rats. No significant alterations were observed in normal rats treated with vanadium complex.

Table 3 depicts the blood urea and plasma creatinine levels and activities of GOT and GPT in normal and experimental groups of rats. The increased levels of urea and creatinine and increase in the activities of these enzymes in diabetic rats were restored to near normalcy in vanadium complex–treated diabetic rats. No significant changes were found in vanadium complex–treated control rats.

**TABLE 2**
Levels of blood glucose, plasma cholesterol, total proteins, and urine sugar in normal and experimental groups of rats

| Group | Blood glucose (mg/dL) | Cholesterol (mg/dL) | Proteins (g/dL) | Urine sugar |
|---|---|---|---|---|
| Normal control | 90 ± 6.1 | 86 ± 3.1 | 7.3 ± 0.3 | Nil |
| Vanadium control | 82 ± 5.9 | 80 ± 2.8* | 7.1 ± 0.5 | Nil |
| Diabetic control | 257 ± 18.9* | 192 ± 5.1* | 5.1 ± 0.8* | +++ |
| Diabetic + vanadium-treated | 99 ± 7.6* | 95 ± 3.3* | 6.8 ± 0.3* | Nil |

*Note*. Values are given as mean ± SD for groups of 6 animals in each group.
Values are statistically significant at $^*P < .05$. Vanadium-treated control rats were compared with control rats. Diabetic control rats were compared with control rats. Vanadium-treated diabetic rats were compared with diabetic control.
+++ indicates more than 2% sugar.

### DISCUSSION

The mononuclear or binuclear oxovanadium complexes so far tested for their hypoglycemic activities contain only acyclic ligands and hence the leaching or hydrolysis of vanadyl ions from these complexes is much easier than in mononuclear or binuclear complexes having macrocyclic ligands, which are relatively more stable. This has encouraged us to develop the macrocyclic binuclear oxovanadium complexes whose hypoglycemic and nontoxic nature are revealed in the present study.

The enhancement of body weight in the vanadium-administered group of rats may be correlated with the observation that vanadium administration enhances the growth up to 40% by increasing the metabolic activity of the system when compared to control rats [31]. This indicates that vanadium increases glucose metabolism and thus enhances body weight in STZ-diabetic rats.

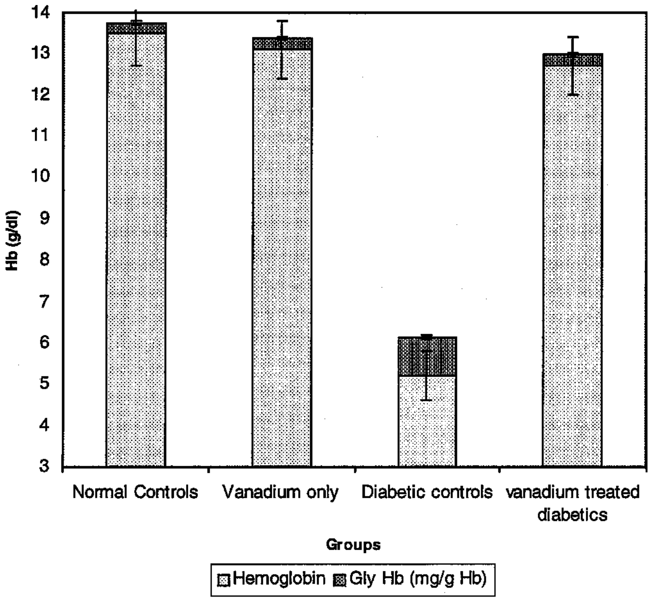

**FIGURE 3**
Levels of Hb and Gly Hb in normal and experimental groups of rats.

Vanadyl ions were reported to exert a marked glucose tolerance after a glucose load in STZ-diabetic rats [32]. Vanadyl ion, owing to its wide range of action by inducing insulin receptor tyrosine kinases and by inhibiting phosphotyrosine phosphatases, enhances the basal rates of glucose uptake and presumed metabolism of glucose by liver and muscle [20]. This results in reverting of hyperglycemic state to normoglycemic state after a glucose load in STZ-diabetic rats. Further, the macrocyclic vanadium complex enhances glucose metabolism by regularizing the activity of glucose-metabolizing enzymes, such as hexokinase, glycogen phosphorylase, and glycogen synthase [33], which resulted in decrease in blood glucose levels in vanadium complex–administered diabetic rats. This may be the reason for marked glucose tolerance exhibited by the vanadium complex to glucose load and thus alleviates diabetes in STZ-diabetic rats treated with macrocyclic vanadium complex.

Obesity is assumed to be a risk factor for insulin resistance and hence is a major cause of non–insulin-dependent diabetes mellitus. In addition to its influence on glucose homeostasis and carbohydrate metabolism, vanadium also plays a major role in lipid metabolism, restoring the altered plasma lipids and lipoprotein profile in STZ-diabetes to near normal levels [34]. Further, by regulating the key regulatory enzyme 3-hydroxy-3-methyl glutaryl coenzyme A synthase, the plasma cholesterol level is regulated by vanadium in STZ-diabetic rats [35]. Thus macrocyclic vanadium complex alleviates insulin resistance by high plasma cholesterol in diabetic rats.

In uncontrolled or poorly controlled diabetes, there is an increased glycation of a number of proteins, including hemoglobin and $\alpha$-crystalline of lens [36]. Glycated hemoglobin ($HbA_1C$) was found to increase in patients with

**TABLE 3**
Levels of blood urea, plasma creatinine, and activities of plasma glutamate oxaloacetic transaminase and glutamate pyruvic transaminases in normal and experimental groups of rats

| Group | Urea | Creatinine | GOT | GPT |
|---|---|---|---|---|
| Normal control | 18 ± 2.9 | 0.69 ± 0.03 | 17 ± 1.6 | 13 ± 0.9 |
| Vanadium control | 19 ± 2.5 | 0.61 ± 0.02* | 18 ± 0.6 | 12 ± 0.8 |
| Diabetic control | 38 ± 3.1* | 0.98 ± 0.04* | 25 ± 1.1* | 26 ± 1.1* |
| Diabetic + vanadium-treated | 20 ± 2.2* | 0.58 ± 0.03* | 16 ± 0.8* | 16 ± 1.2* |

*Note.* Values are given as mean ± SD for groups of 6 animals each.

Values are statistically significant at $^*P < .05$. Vanadium-treated control rats were compared with control rats. Diabetic control rats were compared with control rats. Vanadium-treated diabetic rats were compared with diabetic control.

Activities are expressed as: urea (mg/dL); creatinine (mg/dL); GOT, GPT (n moles of pyruvate liberated/h/mg of protein).

diabetes mellitus to approximately 16% and the amount of increase is directly proportional to the fasting blood glucose level [37]. The decreased level of total hemoglobin in diabetic rats is mainly due to increased formation of glycated hemoglobin [38], which is reverted back to normal levels by the administration of vanadium complex. Vanadate restores the normal levels of glycated hemoglobin in STZ-induced diabetes by its normoglycemic effect [39]. The macrocyclic vanadium complex also controls the glycation of hemoglobin by virtue of its normoglycemic activity.

During diabetes, there is increased protein catabolism, with flow of amino acids into the liver, which feeds gluconeogenesis [40]. Dighe and colleagues [41] have reported that accelerated proteolysis of uncontrolled diabetes occurs as a result of deranged glucagon-mediated regulation of cAMP formation in insulin deficiency. This accounts for the observed decrease in the total protein content in STZ-induced diabetic rats. Administration of vanadium complex to diabetic rats significantly inhibits proteolysis caused by insulin deficiency and thus increases the level of plasma proteins to near normalcy.

Because early reports have claimed that vanadium is toxic to mammals, we undertook a few experiments to elucidate this issue. Morris and Leon [42] reported that increased urea production in diabetes might be due to enhanced catabolism of liver and plasma proteins. However, in the present study, the vanadium complex treated diabetic rats showed near normal values in urea and creatinine levels, which reflects the non-toxic nature of the vanadium complex. It is a well-known fact that vanadium tends to be stored in the kidney, which is the primary storage site under normal conditions. Further, kidney is one of the important target organs of vanadium toxicity [43, 44]. No significant alterations in plasma urea levels in vanadium-treated control and diabetic rats indicate normal kidney function and thus reveal the nontoxic nature of macrocyclic binuclear oxovanadium complex used in the present study. Also, a rise in GPT activity is almost always due to hepatocellular damage followed by cardiac tissue damage and is usually accompanied by a rise in GOT activity [45]. The increase in the activities of GOT and GPT is also found in diabetic BB rats [46]. The reversal of GPT and GOT activities in vanadium complex–treated diabetic rats towards near normalcy further strengthens the nontoxic nature of the complex.

Further in-depth studies on the mechanism of action of this macrocyclic binuclear oxovanadium complex would render us with an efficient nontoxic hypoglycemic drug in the near future.

### REFERENCES

[1] Macara, I. G. (1980) Vanadium: An element in search of a role. *Trends. Biochem. Sci.,* **5,** 92–94.

[2] Srivastava, A. K. (1995) Potential use of vanadium compounds in the treatment of diabetes mellitus. *Exp. Opin. Invest. Drugs.,* **4,** 525–536.

[3] Brichard, S. M., and Henquin, J. C. (1995) The role of vanadium in the management of diabetes. *Trends Pharmacol. Sci.,* **16,** 265–270.

[4] Poucheret, P., Subodh, V., Grynpas, M. D., and McNeill, J. H. (1998) Vanadium and diabetes. *Mol. Cell. Biochem.,* **188,** 73–80.

[5] Schechter, Y., and Karlion, S. J. D. (1980) Insulin-like stimulation of glucose oxidation in rat adipocytes by vanadyl (IV) ions. *Nature,* **284,** 556–558.

[6] Shafrir, E., Spielman, S., Nachliel, I., Khamaisi, M., Bar-On, H., and Ziv, E. (2001) Treatment of diabetes with vanadium salts: General overview and amelioration of nutritionally induced diabetes in the Psammomys obesus gerbil. *Diabetes Metab. Res. Rev.,* **17,** 55–66.

[7] Dubyak, G. R., and Kleinzeller, A. (1980) The insulin-mimetic effects of vanadate in isolated rat adipocytes. *J. Biol. Chem.,* **255,** 5306–5312.

[8] Degani, H., Gochin, M., Karlish, S. J. D., and Schechter, Y. (1981) Electron paramagnetic studies and insulin-like effects of vanadate in rat adipocytes. *Biochemistry,* **20,** 5795–5799.

[9] Schechter, Y. (1990) Perspectives in diabetes, insulin-mimetic effects of vanadate. Possible implications for future treatment of diabetes. *Diabetes,* **39,** 1–5.

[10] Heyliger, E. C., Tahilini, G. A., and McNeill, H. J. (1985) Effect of vanadate on elevated blood glucose and depressed cardiac performance of diabetic rats. *Science,* **227,** 1474–1476.

[11] Ramanadham, S., Mongold, J. J., Brownsey, R. W., Cors, G. H., and McNeill, J. H. (1989) Oral vanadyl sulfate in treatment of diabetes mellitus in rats. *Am. J. Physiol.,* **257,** H904–H911.

[12] Sakurai, H., Tsuchiya, K., Nukatsuka, M., Kawada, J., Ishikawa, S., Yoshida, H., and Komatsu, M. (1990) Insulin-mimetic action of vanadyl complexes. *J. Clin. Biochem. Nutr.,* **8,** 193–200.

[13] McNeill, J. H., Yuen, V. G., Hoveyda, H. R., and Chris, O. (1992) Bis- (maltolato) oxovanadium (IV) is a potent insulin mimic. *J. Med. Chem.,* **35,** 1489–1491.

[14] Nandhini, D., Maneemegalai, S., Elangovan, V., Sekar, N., and Govindasamy, S. (1993) Insulin like effects of bis-glycinato oxovanadium (IV) complex on experimental diabetic rats. *Indian J. Biochem. Biophys.,* **30,** 73–76.

[15] Watanabe, H., Nakai, M., Komazawa, K., and Sakurai, H. (1994) Bis-pyrrolidin-*N*-carbodithiato oxovanadium (IV) is a potent insulin mimic. *J. Med. Chem.,* **37,** 876–877.

[16] Sekar, N., Li, J., and Schechter, Y. (1996) Vanadium salts as insulin substitutes: Mechanism of action, a scientific and therapeutic tool in diabetes mellitus research. *Crit. Rev. Biochem. Mol. Biol.,* **31,** 339–359.

[17] Domingo, J. L., Llobet, J. M., Tomas, J. M., and Corbella, J. (1985) Short term toxicity studies of vanadium in rats. *J. Appl. Toxicol.,* **5,** 418–421.

[18] Shanchez, D. J., Ortega, A., Domingo, J. L., and Corbella, J. (1991) Developmental toxicity evaluation of sodium orthovanadate in the mouse. *Biol. Trace Elem. Res.,* **30,** 219–229.

[19] Nanda, K. K., Mohanta, S. K., Ghosh, S., Monika, M., Helliwell, M., and Nag, K. (1995) Macrocyclic mononuclear $V^{IV}$ and $V^{V}$, heterodinuclear $V^{V}$ $Ni^{V}$ and heterodinuclear $V^{IV}$ $Ni^{II}$ $V^{V}$ complexes: Synthesis, structure, electrochemistry and magneto chemistry. *J. Inorg. Chem.,* **34,** 2861–2869.

[20] Meyerovitch, J., Farfel, Z., Sack, J., and Schechter, Y. (1987) Oral administration of vanadate normalizes blood glucose levels in streptozotocin treated rats. *J. Biol. Chem.,* **262,** 6658–6662.

[21] Sasaki, T., Matsy, S., and Sonae, A. (1972) Effect of acetic acid concentration on the colour reaction in the *O*-toluidine boric acid method for blood glucose estimation. *Rinsh. Kagaku.,* **1,** 346–353.

[22] Natelson, S., Scott, M. L., and Begga, E. (1951) A rapid method for the estimation of urea in biological fluids by means of the reaction between diacetyl and urea. *Am. J. Clin. Pathol.,* **21,** 275–281.

[23] Drabkin, D. L., and Austin, J. M. (1932) Spectrophotometric constants for common hemoglobin derivatives in human, dog and rabbit blood. *J. Biol. Chem.,* **98,** 719–733.

[24] Nayak, S. S., and Pattabiraman, T. N. (1981) A new colorimetric method for the estimation of glycosylated hemoglobin. *Clin. Chem. Acta,* **109,** 267–274.

[25] Zlatkis, A., Zak, B., and Boyle, A. J. (1953) Method for the determination of serum cholesterol. *J. Clin. Medicare,* **41,** 486–490.

[26] Brod, J., and Sirota, J. H. (1948) Renal clearance of endogenous creatinine in man. *J. Clin. Invest.,* **27,** 645.

[27] Lowry, O. H., Rosenbrough, N. J., Farr, A. L., and Randall, R. (1951) Protein determination using Folin-Ciocalteu Reagent. *J. Biol. Chem.,* **193,** 265.

[28] King, J. (1965) The transferase-alanine and aspartate transaminase. In: *Practical Clinical Enzymology*, Edited by Princeton, M. J., pp. 363–395. London, Van D Wostrand.

[29] Whittington, K. B., Solomon, S. S., and Lu, Z. N. (1991) Islet allograft in the cryptorchid testes of spontaneously diabetic BB/W or dp rats: Response to glucose, glipizide and arginine. *Endocrinology,* **128,** 2671–2677.

[30] Pari, L., and Umamaheswari, J. (1999) Antihyperglycaemic activity of *Musa sapeintum L.* in alloxan-induced diabetic rats. *J. Ethnopharmacol.,* **68,** 1–5.

[31] Schwarz, K., and Milne, D. B. (1971) Growth effects of vanadium in the rat. *Science,* **174,** 426–428.

[32] Sakurai, H., Tsuchiya, K., Nukatsuka, M., Sofue, M., and Kawada, J. (1990) Insulin-like effect of vanadyl ion on streptozotocin-induced diabetic rats. *J. Endocrinol.,* **126,** 451–459.

[33] Ramachandran, B., Kandasamy, M., Narayanan, V., and Subramanian, S. (2003) Insulin mimetic effects of macrocyclin binuclear oxovanadium complexes on STZ-induced experimental diabetes in rats. *Diabetes Obesity Metab.,* **5,** 455–461.

[34] Sekar, N., and Govindasamy, S. (1991) Effects of vanadate on plasma lipoprotein profiles in experimental diabetic rats. *Biochem. Int.,* **23,** 935–940.

[35] Valera, A., Rodriguez-Gil, J. E., and Bosch, F. (1993) Vanadate treatment restores the expression of genes for key enzymes in the glucose and ketone bodies metabolism in the liver of diabetic rats. *J. Clin. Invest.,* **92,** 4–11.

[36] Alberti, K. G. M. M., and Press, C. M. (1982) The biochemistry and the complications of diabetes. In: *Comlications of Diabetes*, Edited by Keen, H., and Jarre, J., pp. 231–270. Edward Arnold, London.

[37] Koenig, R. J., Peterson, C. M., Jones, R. L., Saudek, C., Lehrman, M., and Cerami, A. (1976) Correlation of glucose regulation and hemoglobin A1 C in diabetes mellitus. *N. Engl. J. Med.,* **295,** 417–420.

[38] Venkateswaran, S., and Pari, L. (2002) Effect of Coccinia indica on blood glucose, insulin and hepatic key enzymes in experimental diabetes. *Pharmacol. Biol.,* **40,** 165–170.

[39] Sekar, N., Kanthasamy, S., William, S., Subramanian, S., and Govindasamy, S. (1990) Insulinic action of vanadate on experimental diabetes. *Pharmacol. Res.,* **22,** 207–217.

[40] Rannels, D. E., Mokee, D. E., and Morgan, H. E. (1997) Action of insulin. In: *Biochemical Actions of Hormones*, Edited by Lit Wack, G., pp. 135–195. New York, Academic Press.

[41] Dighe, R. R., Rojas, F. J., and Birnbaumer, L. (1984) Glucagon stimulates adenylate cyclase in rat liver. The impact of STZ-induced diabetes mellitus. *J. Clin. Invest.,* **73,** 1013–1024.

[42] Morris, G., and Leon, L. M. (1960) Protein metabolism and protein synthesis in perfused livers of normal and alloxan diabetic rats. *J. Biol. Chem.,* **235,** 3202–3208.

[43] Hansen, T. V., Aaseth, J., and Alexander, J. (1982) The effect of chelating agents on vanadium distribution in rat body and on uptake by human erythrocytes. *Arch. Toxicol.*, 50, 195–198.

[44] Jandhyala, B. S., and Hom, G. J. (1983) Physiological and pharmacological properties of vanadium. *Life Sci.,* **33,** 1325–1331.

[45] Rao, G. M., Morghom, L. O., Kabur, M. N., Ben Mohmud, B. M., and Ashibani, K. (1989) Serum glutamic oxaloacetic transaminase (GOT) and glutamic pyruvic transaminase (GPT) levels in diabetes mellitus. *Indian J. Med. Sci.,* **43,** 118–121.

[46] Scott, F. W., Trick, K. D., Lee, L. P., Hynie, I., Heick, H. M., and Nera, E. A. (1984) Serum enzymes in the BB rat before and after onset of the overt diabetic syndrome. *Clin. Biochem.,* **17,** 270–275.

